# A pressure driven electric energy generator exploiting a micro- to nano-scale glass porous filter with ion flow originating from water

**DOI:** 10.1038/s41598-022-21069-8

**Published:** 2022-10-20

**Authors:** Yo Tanaka, Satoshi Amaya, Shun-ichi Funano, Hisashi Sugawa, Wataru Nagafuchi, Yuri Ito, Yusufu Aishan, Xun Liu, Norihiro Kamamichi, Yaxiaer Yalikun

**Affiliations:** 1 Center for Biosystems Dynamics Research (BDR), RIKEN, 1-3 Yamadaoka, Suita, Osaka 565-0871 Japan; 2 Department of Robotics and Mechatronics, Tokyo Denki University, 5 Senju-Asahi-Cho, Adachi-Ku, Tokyo, 120-8551 Japan; 3 Graduate School of Nara Institute of Science and Technology, 8916-5 Takayamacho, Ikoma, Nara 630-0192 Japan

**Keywords:** Energy harvesting, Devices for energy harvesting, Electrical and electronic engineering, Nanoscience and technology, Electronic devices, Nanofluidics, Nanopores

## Abstract

We demonstrated a pressure driven energy harvesting device using water and that features a glass filter with porous channels. We employed powder sintering to fabricate the glass filter (2 cm diameter, 3 mm thickness) by packing a powder of borosilicate glass particles into a carbon mold and then thermally fusing this at 700°C under pressure. In constant flow rate experiment, the optimum average pore radius of the filter for power generation was 12 μm. Using this filter, power of 3.8 mW (27 V, 0.14 mA, 0.021% energy efficiency) was generated at a water flow speed of 50 mm/s. In constant pressure experiment, a power generator was equipped with a foot press unit with a 60 kg weight (830 kPa) and 50 mL of water. The optimum average pore radius for power generation in this experiment was 12 μm and power of 4.8 mW (18 V, 0.26 mA, 0.017% energy efficiency) was generated with 1.7 s duration. This was enough power for direct LED lighting and the capacitors could store enough energy to rotate a fan and operate a wireless communicator. Our pressure driven device is suitable for energy harvesting from slow movements like certain human physiological functions, e.g. walking.

## Introduction

Energy harvesting is a promising technology to power multiple small electronic devices in the future Internet of things (IoT) society^1^. Light, thermal and mechanical energies are usually used for energy harvesting. Among them, mechanical energy harvesting that obtains electrical energy from mechanical motions of oscillation and vibration is quite familiar to everyone and large amounts of energy can be obtained^2,3^. For instance, electromagnetic induction devices^4–6^, piezoelectric (electromechanical material) devices^7–11^ and electrostatic devices^12–14^ have been developed and used. However, miniaturized electromagnetic induction devices generally have low efficiency which is undesirable. Piezoelectric devices or electrostatic devices can be miniaturized, but the conversion efficiency is decreased when the vibration frequency is small (e.g. less than 10 Hz). Therefore, it is difficult for these devices to fully exploit a human being’s regular slow physiological motions, e.g. walking.

In this paper, we focus on the phenomena occurring in a pressure driven electric energy generation device by the interaction between pure water and a surface charged solid (a glass filter). The detailed principle is explained below. This approach is useful for small frequency vibrations because energy generation continues as long as water exists in the device. There have been some reports on power generation devices based on this principle that use glass coated silicon^15^, metal–carbon composite^16^, or cellulose^17^. However, it is difficult to apply a high pressure to these materials due to their fragility, and therefore the power that they can generate is not so high.

On the other hand, glass is hard and robust and a high pressure can be applied to it. There have been reports of water pressure driven power generation using glass channels made by top-down micro or nano fabrication methods^18–21^. However, such power generation devices are not powerful enough for energy harvesting because the generated current per channel is generally very small (picoampere order).

We used porous glass to increase the pore channel numbers based on an investigation and optimization of the device fabrication process. Although porous glass was previously used for power generation^22^, the power is still low because the fabrication of a porous glass filter has not been optimized for power generation. Numerous technologies exist to fabricate many kinds of glass based microfluidic devices^23^. Based on these concepts and technologies, our aim in this study was to investigate the pore size effect on the generation performance, and to develop an actual energy harvesting device consisting of the porous glass filter and foot press unit to transfer the force of an experimenter’s foot pressing to the power generator to demonstrate actual energy harvesting.

A similar power generation method, osmotic power generation by nano pore membranes, is well known^24^. That method extracts power from mixing salt solutions of different concentrations using a nano porous material including carbon^25^, aluminum oxide^26^, or silicon nitride^27^. The important difference is that the osmotic power generation requires an ion concentration gradient and external pressure cannot be used.

## Principle

**Figure 1:**
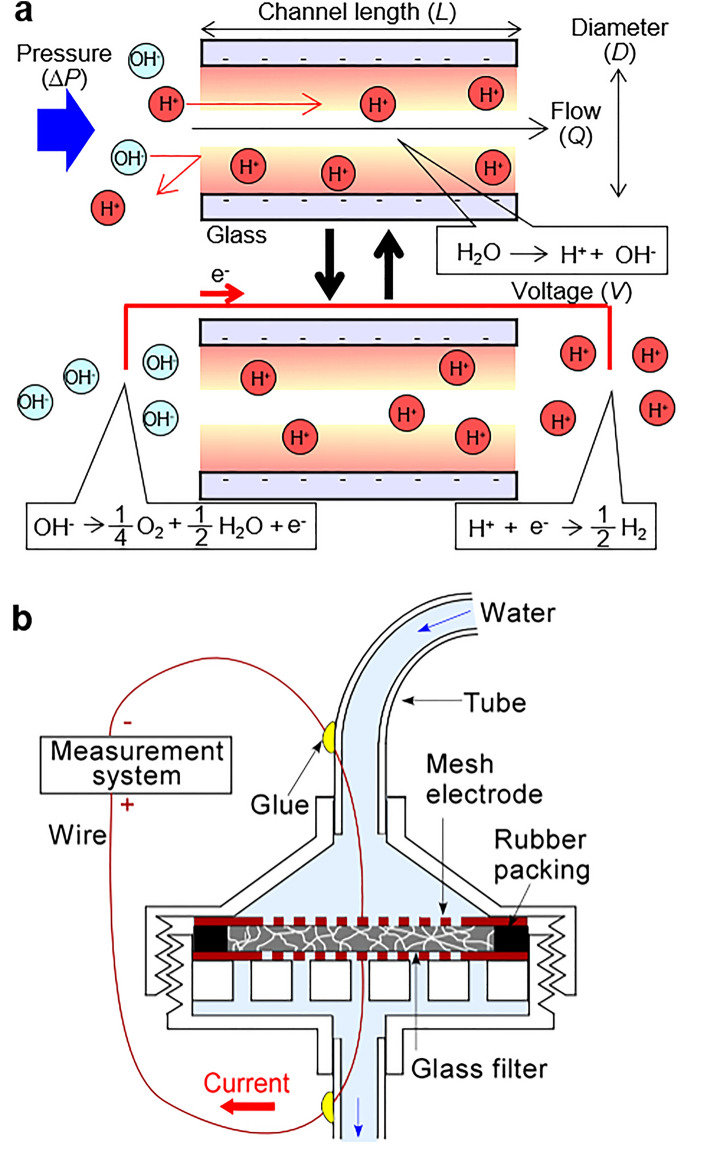
Design and principle of the pressure driven electric power generator using a porous glass filter. (**a**) Principle of electric generation by pressure in a micro- to nano-scale glass channel. The phenomena for these top and bottom schematics occur at the same time. Parameters used in Eqs. (1) and (2) are also defined. (**b**) Cross-sectional design of the prototype power generator.

The principle of the electric power generation by water pressure is shown in Fig. 1a. It is supposed that pure water flows into the nano- or micro-sized glass channels. Water molecules are partially dissociated by thermal balance and ionized to protons (H^+^) and hydroxyl ions (OH^−^). When water is introduced into small channels, it is easy for H^+^ that is produced to enter the channels, but it is not easy for OH^−^ to enter them. This is because the glass channel surface is negatively charged due to silanol groups on the surface after H^+^ species are detached into the water. As a result, the H^+^ concentration increases in the outlet while the OH^−^ concentration increases in the inlet. Namely, the glass channel works as an ion filter if the channel size is very small.

In this situation, electrical energy transfer occurs by electronically connecting the inlet and outlet by a wire. At the inlet electrode, OH^−^ forms oxygen and water molecules, and produces electrons. At the outlet electrode, H^+^ takes up electrons and forms hydrogen molecules. Current flows by these reactions. After the ions react and are consumed in the gas molecule generations, new ions are produced from the remaining water to maintain thermal balance. If the produced hydrogen and oxygen gas molecules can be recombined as water by a catalytic reaction, this reaction cycle can occur continuously, and also the gases might be used as energy source. Actually, the amounts of hydrogen and oxygen gases generated are not much, (typically under a nanogram order for a reaction cycle occurring for several minutes). Therefore, in this report, we used electrical power generated by an ion filter.

Theoretically, the generation voltage (*V*) is described by Eq. (1), the Helmholtz-Smoluchowski equation^28^:

1 $$V = \, ( \varepsilon \xi /\mu K)\Delta P$$

where *ε* is permittivity, *ξ* is zeta potential, *µ* is viscosity, *K* is water conductivity, and *ΔP* is applied pressure. Since *ε*, *ξ*, *µ* and *K* are fixed parameters decided by the surface and water properties and not related to channel geometry, *V* is simply in proportion to *∆P*. Also, *ΔP* is described by Eq. (2), the Hagen–Poiseuille law^29^:

2 $$\Delta P = \, ({128}\mu L/\pi D^{4} )Q$$

where *D* is the relative channel diameter, *L* is the channel length, and *Q* is flow rate. From Eqs. (1) and (2), it is clear that *D* must be smaller to improve the voltage if *Q* is constant. To improve the power generation performance, parallel smaller channels should be designed with an as large as possible area. In this context, using porous glass is a rational design choice.

Figure 1b shows the design of a prototype power generator device. A rubber packing ring was used to tightly fit a porous glass filter (2 cm in diameter and 3 mm in thickness) into a commercially available filter holder that had been modified slightly (e.g. the opening for tubing attachment was shaved to enlarge it). A copper mesh electrode was placed above and below the glass filter. The inlet and outlet tubes were attached using epoxy glue at the places indicated in the figure.

## Results

### Investigation of sintering temperature conditions for glass filter fabrication

**Figure 2:**
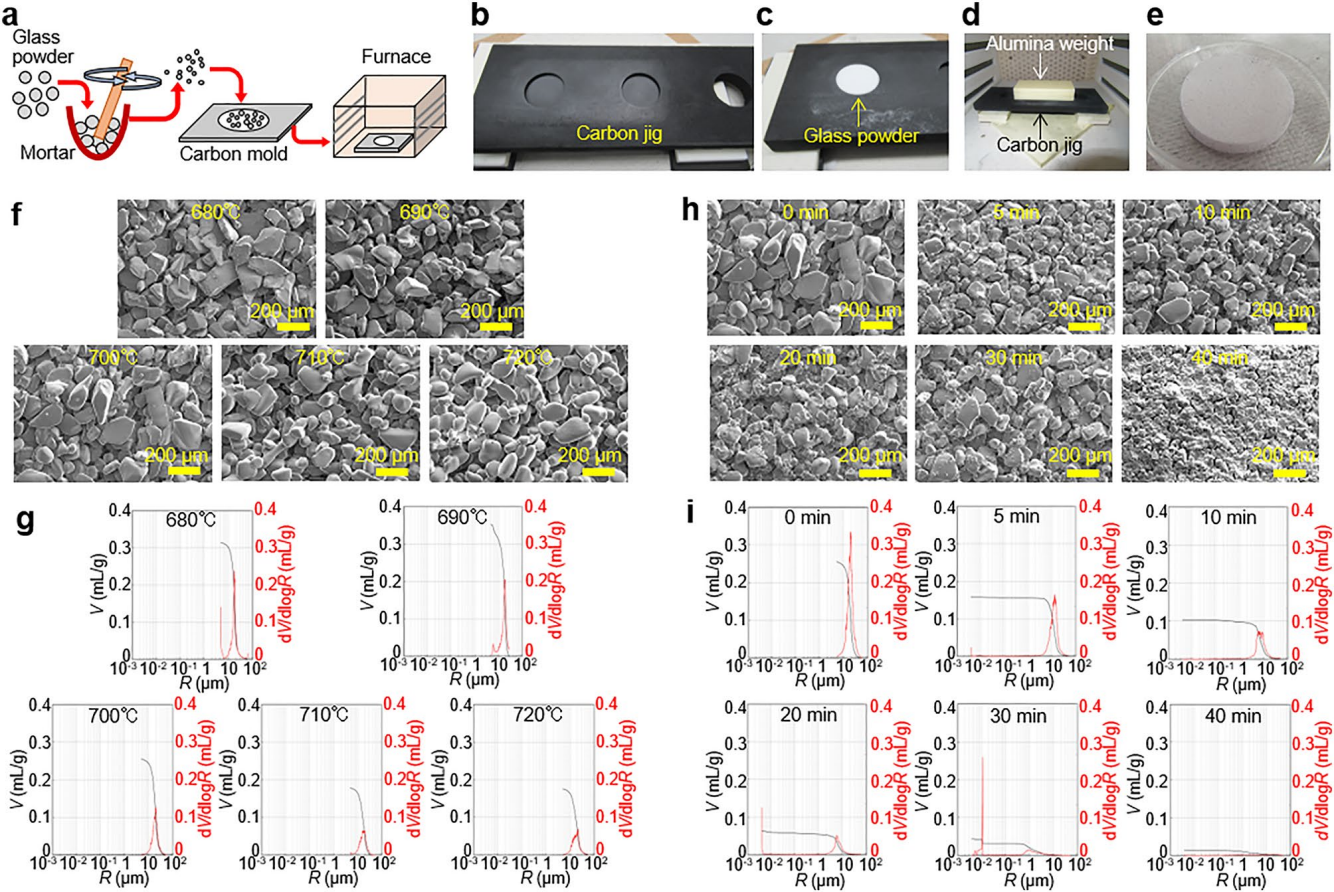
Fabrication and structure investigation of porous glass filters. (**a**) Fabrication procedure of glass filters. (**b**) Carbon mold to fabricate glass filters. (**c**) A hole in the mold is filled with glass powder. (**d**) Setup in a furnace with an alumina weight. (**e**) Fabricated glass filter. (**f**) SEM images of porous glass filter surfaces after sintering at the temperature indicated at the top of each image. (**g**) Results of mercury porosimetry of the porous glass filters sintered at various temperatures. Black and red lines indicate pore volume per mass (*V*) and pore radius (*R*) distribution (derivation of *V* by *R*), respectively. (**h**) SEM images of the porous glass filter surfaces sintered at 700 °C using ground glass powder and the milling time indicated at the top of each image. (**i**) Results of mercury porosimetry of the porous glass filters sintered at 700 °C using ground glass powder and the milling time indicated at the top of each image. Scale bars are shown in SEM images of (**f**) and (**h**).

We first established the fabrication process of porous glass filter and then investigated the structure for various fabrication conditions. The fabrication details are described in Fig. 2a and the Methods section. We employed powder sintering by packing a powder of borosilicate glass particles in a carbon mold and thermally fusing this under pressure applied from a weight as shown in Fig. 2b–d. Although laser fabrication^30–32^ is commonly used to make glass filters, sintering is simpler and provides robust filters with large numbers of channels. A borosilicate glass filter plate (diameter of 2 cm) was fabricated (Fig. 2e). Normally for tight bonding by glass thermal fusion, a temperature of 750 °C is used^33,34^. However, at this temperature, the borosilicate glass particles completely melted, and became gray colored and no water could pass through it. Based on our previous experiences^35,36^ glass and glass can be bonded to each other if pressure is applied even at a lower temperature and we used the sintering temperatures of 680–720 °C here. Under these conditions, we obtained good glass filters with no discoloration due to degradation. However, observation with a microscope showed that the edge of the glass particles was slightly melted at 710 and 720 °C (Fig. 2f). Also, the filter was rather fragile when it was sintered at 680 and 690 °C.

The pore size distribution was measured by the mercury porosimetry for filters prepared at various sintering temperatures and the results are plotted in Fig. 2g. The peak in the pore size distribution was not so different at different temperatures. At all temperatures, the peak was at 20 μm (average pore radius). But the peak height decreased when the temperature was higher. This meant that the pores were filled with melted glass when the sintering temperature was increased. Considering the findings and to avoid fragility, we concluded that the optimum sintering temperature was 700 °C. We used this condition to investigate the effect of particle size in the glass filter fabrication next.

### Investigation of glass particle milling condition for glass filter fabrication

To control the glass particle size, particles were milled. The scanning electron microscope (SEM) images and diameter histograms of milled particles with various milling times are shown in Fig. S1. The average Feret diameter, defined as the distance between two parallel planes restricting the object perpendicular to that direction, could be controlled between 4 and 150 μm.

The milled particles were used to fabricate glass filters with sintering at 700 °C. From the SEM images (Fig. 2h), we saw that most of the particles maintained their shapes, although small particles were observed to be partially melted especially at longer milling times. This was consistent with the pore size distribution results (Fig. 2i). At 0, 5, 10, 20 and 30 min milling times, there were peaks at 20, 12, 8, 5 and 1 μm (average pore radius), respectively. At the 40 min milling time, it was difficult to find a peak. This indicated that the pore size became small when milling time was increased, but the pore volume (peak height) decreased due to the melting of the small particles. Especially at milling times of 30 and 40 min, the pore sizes were nanometer scale and the peak height was very small. These properties are deeply related with the electric power generation performance which we investigated next.

### Demonstration of the pressure driven electric power generator

**Figure 3:**
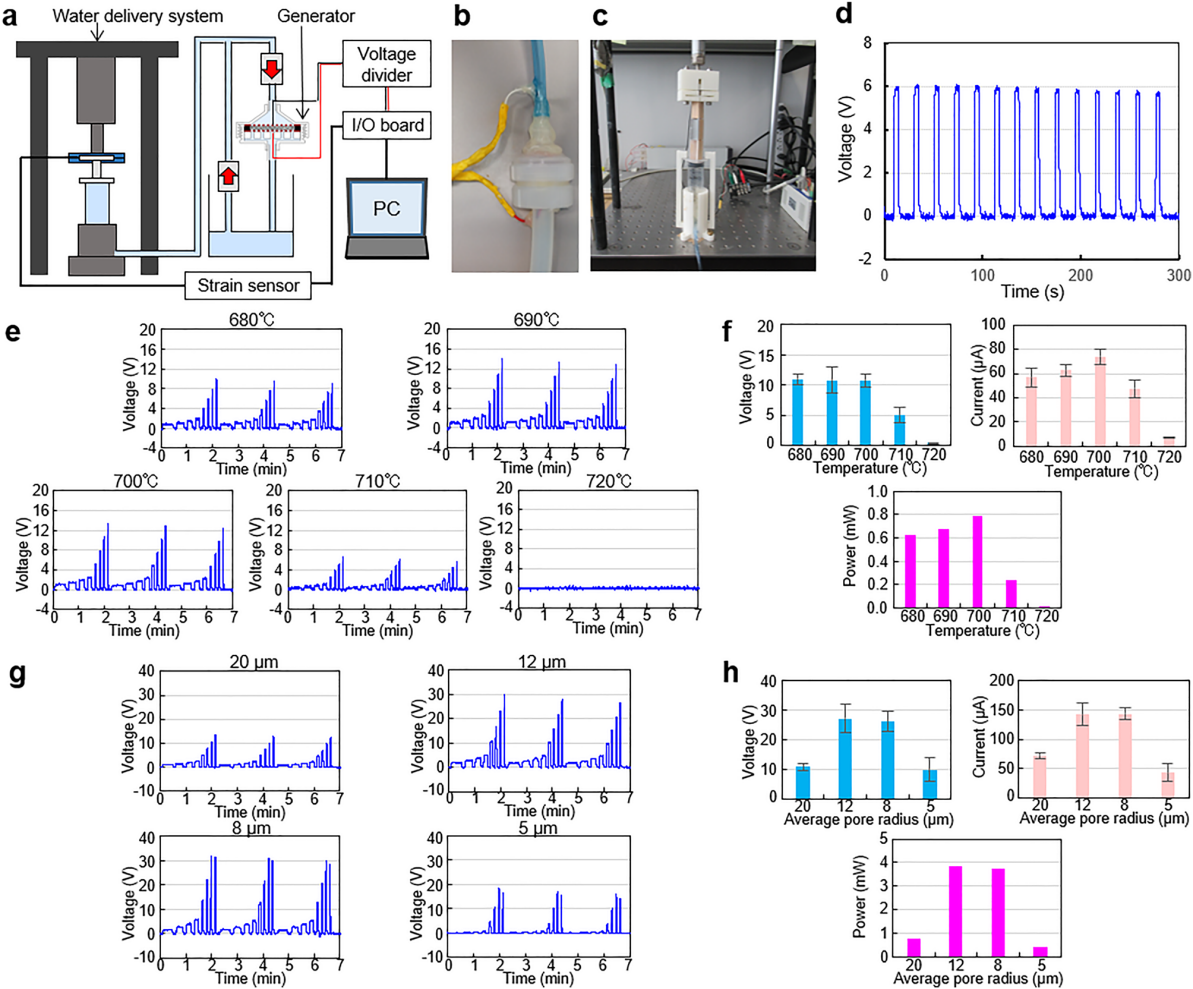
Characterization of power generating performance of fabricated glass filters. (**a**) Setup for characterization of the glass filters. Water is introduced into the generator by the water delivery system and it is circulated using check valves. Measured voltage is recorded by a PC through an I/O board. (**b**) Photo of the generator equipped with the glass filter and electrodes. (**c**) Photo of the water delivery system. (**d**) Voltage time-course of the repetitive power generation using the filter sintered at 700 °C and 5 min glass powder milling time. The water delivery system speed was 20 mm/s. (**e**) Voltage time-courses using the porous glass filters sintered at the temperature indicated at the top of each graph. Each graph shows voltage during 3 pressing cycles at water delivery system speeds of 4, 6, 8, 10, 20, 30, 40 and 50 mm/s. (**f**) Voltage, current and estimated power of the generators versus sintering temperature of the glass filters at the water delivery system speed of 50 mm/s. Voltage and current plots represent average ± S.D. (n = 3). (**g**) Voltage time-courses using the porous glass filters sintered at 700 °C with average pore radius indicated at the top of each graph. Water delivery conditions were the same as in (**e**). (**h**) Voltage, current and estimated power of the generators versus average pore radius. Voltage and current plots represent average ± S.D. (n = 3).

Using the fabricated porous glass filters, we demonstrated the electric power generator and investigated the pore size effect using a constant speed water delivery system (Figs. 3a–c). The power generator was constructed by setting a glass filter in a modified commercial filter holder and inserting the electrodes above and below the filter (Fig. S2). All experiments using the water delivery system were carried out at room temperature. Deionized pure water produced using a Milli-Q system was introduced into the generator, and a voltage was generated (Fig. 3d). Repetitive voltage generation was also confirmed. A slight voltage decrement was observed, but this was probably because we recycled the water. The mesh number and the distance between the glass filter and the mesh electrode were optimized and these results are summarized in Fig. S3.

As show in Fig. 3e and f, the voltage was in proportion to the speed of the water flow. At the same speed, voltage generation performance was almost equal using the filters sintered at 680, 690 and 700 °C, but it decreased for filters sintered at 710 and 720 °C. This meant that the small pores disappeared at higher sintering temperatures. The current was measured from the slope of the voltage saved in a capacitor during the water flowing (Fig. S4). This generated current had the similar tendency as the voltage, although the peak performance was seen for the filter sintered at 700 °C. This was simply because the number of small pore size increased with temperature increment up to 700 °C, but at higher temperatures the pores were almost completely closed. The voltage, current and power calculated by the product of voltage and current had peaks (11 V, 74 μA and 0.80 mW, respectively) for the filter sintered at 700 °C. From this result, we judged the sintering temperature of 700 °C gave the optimum filter for the power generator.

Then, the filters made from milled particles sintered at 700 °C were used for the power generation experiment. As show in Fig. 3g and h, the filter made using the 5 min milling time (12 µm average pore radius) provided the peak voltage, current and power values of 27 V, 0.14 mA and 3.8 mW, respectively.

### Evaluation of the pressure driven electric power generator

We also analyzed the power generator performance in detail using the force measurement results to evaluate the data validity of the water pressure based generation method. The force and pressure applied to the syringe by the water delivery system was measured by a force transducer and the results are shown in Fig. S5. The force was generally in proportion to the speed and filter fineness.

From these results, it was reasonable that the generated voltage increased for filters fabricated with no milling (20 μm average pore radius) to 5 min milling time (12 μm average pore radius) in accordance with the increment of pressure according to Eqs. (1) and (2), but the generated voltage decreased for filters fabricated with longer milling times due to closing of the pores. Especially for the milling times of 30 min or longer (5 μm or smaller average pore radius), the filters could not withstand the pressure and they broke during the experiment. So, we had no data for these conditions. Another reason may be that the electric properties of water in sub-microscale channels are different from those in a bulk space^37,38^. Considering an applied force of 350 N to the water and the syringe pushing speed (50 mm/s), the peak power efficiency using a filter fabricated with 5 min milling time (12 μm average pore radius) was calculated as 0.021%.

### Verification experiment using a fused silica filter

Although the power generator was verified in the previous section, we were concerned that the current was generated by impurities included in the borosilicate glass. To confirm that the generation was caused only by the interaction between the glass surface and water, we used fused silica particles which included almost no impurities for the filter. The result is summarized in Fig. S6. The fabricated fused silica filter is shown in Fig. S6b. Although this filter was more fragile than the borosilicate glass filters, it could be used for a power generation experiment at low water flow speed of less than 20 mm/s. The repetitive voltage generation was confirmed, and water flow was smooth as shown by the force measurement data (Fig. S6d). From these results, we confirmed that the electric power generation was not caused by impurities in the borosilicate glass.

### Demonstration of power generation device with a foot press unit

**Figure 4:**
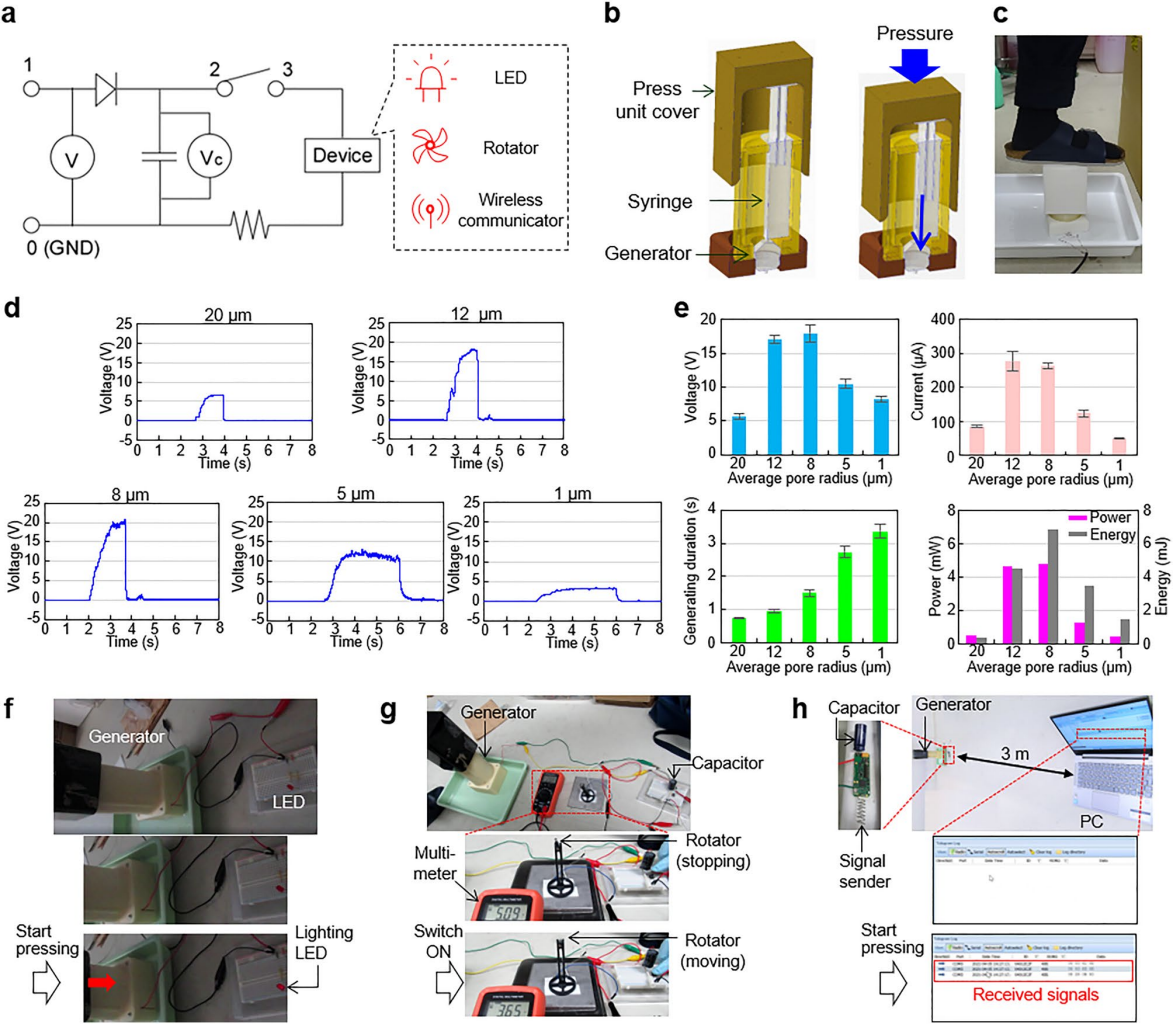
Demonstration and application of the electric power generator equipped with a foot press unit. (**a**) A circuit for capacitor storage and device driving applications. A generator was connected to ports 0 and 1. For energy saving applications, the generator was used without connecting ports 2 and 3. (**b**) Schematic drawings showing the working motion of the foot press unit. (**c**) Photo of the foot press unit. (**d**) Voltage time-courses using porous glass filters sintered at 700 °C with ground glass powder for the average pore radius indicated at the top of each graph. Each graph shows the voltage during 1 pressing cycle using a 60 kg weight (foot pressing by an experimenter) starting at about 2 s. (**e**) Voltage, current, power generating duration, estimated power and energy of the generator versus average pore radius. Voltage and current plots represent average ± S.D. (n = 3). (**f**) Application of direct LED lighting by connecting the LED to the generator (without a switch and capacitor). The upper photo shows the overall setup and the lower 2 photos are before and after foot pressing. (**g**) Application to driving a rotator by saving energy in the capacitor. The upper photo shows the overall setup and the lower 2 photos show the rotator (fan) and the multimeter display screen which gives the accumulated voltage in the capacitor before and after turning the switch on to release the energy in the capacitor. (**h**) Application to a wireless communication tool busing energy stored in the capacitor. After energy accumulation, the communication tool automatically sends a signal to the PC. The top right photo shows the overall setup and the top left photo is an enlarged image of the capacitor and sender. The middle and bottom photos are screen grabs generated by the software to confirm signal receiving and sending.

Following the fundamental investigations of the previous sections, we fabricated a prototype power generation device with a foot press unit as shown in Fig. 4a–c. The power and the continuing duration of the generation were measured. The foot press unit (Fig. 4b) included a syringe and its holder, a holder for the power generator and a cover; and the unit was designed to easily and effectively transfer the applied external force to the water in the syringe. We introduced 50 mL of deionized pure water (Milli-Q water) into the syringe that was set in the unit. A weight of about 60 kg (588 N, corresponding to 830 kPa pressure) was applied when an experimenter pressed on this unit with the foot (Fig. 4c). The electrical measurement and recording method was the same as that for the size effect experiment.

The foot press unit was smoothly driven by the experimenter’s foot pressing motions, and it could be continuously used for at least 100 times during an experiment. The 50 mL of water was repeatedly used by recovering it each time after finishing the pressing motion for up to 50 times in an experiment. Figure 4d and e show the power generation experimental results. The voltage increased immediately after applying the pressure (at *t* = 2–3 s) and dropped to zero when all the water in the syringe was emptied. The generated voltage and current peaked using the filter fabricated with the milling time of 5 min (12 μm average pore radius). This was the same result as obtained for the experiment using the constant speed water delivery system. However, the duration of power generation increased when the milling time increased, because this experiment used a constant pressure for generation (the experimenter’s foot pressing). Conversely, the flow rate decreased when the pore size became smaller (Fig. S7a). So, considering the duration, the graph of harvested energy vs. milling time had a right shifted peak compared to the power graph (Fig. 4e). The voltage, current and power had peaks of 18 V, 0.26 mA and 4.8 mW, respectively, for the filter made with the 10 min milling time (8 μm average pore radius). Since the energy was generated for a duration of 1.7 s for this filter, the harvested energy was 6.8 mJ. Considering the applied force (588 N) and syringe pushing displacement (70 mm), we calculated the peak power efficiency for the filter made with the 10 min milling time (8 μm average pore radius) was 0.017%.

### Application of the pressure driven electric power generator

To demonstrate that the obtained power was able to drive circuits and electronic devices, we carried out a light-emitting diode (LED) lighting test, a fan rotation test and a wireless commination tool test. First, a LED light was illuminated via a direct connection without using a capacitor. The experimental setup is shown in Fig. 4f and Supplementary Movie 1. The pressing time for squeezing out all the stored water from the syringe through the glass filter was roughly 2 s. The LED was lit during this pressing time. This application is easy to understand and could be used for lighting when a person is walking in a dark place.

A mini fan was assembled from a mini motor and other 3D printed parts and in a similar way to the LED lighting was driven as shown in Fig. 4g. But unlike for the LED lighting, more electricity was needed for the fan rotation. Therefore, we used a capacitator with a large capacitance (4700 µF) and the capacitator was charged by 50 foot presses. The stored voltage in the capacitor was measured as 5.2 V. The glass filter power generator produced sufficient electricity to rotate the mini fan. Related experimental results are given in Supplementary Movie 2. We demonstrated that it was feasible to drive something like the mini fan, which could be used for cooling when a person is walking.

Finally, a wireless communication tool was driven as shown in Fig. 4h; such tools are widely used for smart monitoring by constantly sending a signal about the immediate surroundings that includes temperature, light, and motion changes^39^. The signal generator was attached to a capacitor (2200 µF) and it automatically sent the signal after the energy (about 0.2 V) was stored in it. When the foot press unit was stepped on twice, the wireless communication tool transmitted signals to the signal receiver which were monitored by the PC. The actual signal catching motion is shown in Supplementary Movie 3. When a wireless kit composed of a signal generator and a capacitor was powered by the glass filter power generation device, it simultaneously triggered signals and sent them to the receiver. Once the signals were received, their receipt was reflected on the monitor until all the voltage in the capacitor had been consumed. We sent signals over a 3 m distance in this experiment. This application would be practical for personal health monitoring.

### Additional validation

According to the principle of electric generation, hydrogen and oxygen gases are generated. Here, we estimated how much volume was generated. The amount of substance of hydrogen (*n*_*h*_) can be calculated by Eq. (3):

3 $${\text{n}}_{{\text{h}}} = It/{2}F$$

where *I* is the current, *t* is the duration of power generation, and *F* is the Faraday constant (9.6 × 10^4^ C/mol). In the foot press experiment, the largest current (*I*) was 0.26 mA and the corresponding duration (*t*) was 1.7 s. In this condition, *n*_*h*_ was calculated as 24 nmol (48 ng). The amount of substance of oxygen (*n*_*o*_) was half of *n*_*h*_ which was calculated as 12 nmol (380 ng). Since the water volume in this experiment was 50 mL, the concentrations of hydrogen and oxygen were estimated as 0.96 ppb and 7.7 ppb, respectively. Such low concentrations are difficult to be measured even using commercially available gas monitoring devices with high sensitivity. To measure the gases, the current must be increased significantly.

However, it is important to confirm that the current generation was not caused by other reasons (e.g. vibration or noise). Therefore, we have obtained the negative control data. The generated voltage by applying 830 kPa pressure using the press unit without a glass filter in the generator is shown in Fig. S7b. It was 0.12 ± 0.04 V (n = 3, ± S.D.) and significantly smaller than the data with glass filters. From this result, the principle of the generation was confirmed.

In addition, we investigated the influence of flow to the resistance of a glass filter. If the resistance changed significantly by flow, the current might not be measured correctly due to the leak current. The simulation was added in Fig. S8. It shows that the resistance was almost the same regardless of the presence or absence of flow. In addition, the electrical resistance was actually measured using the press unit and the filter with 8 μm average pore radius. The resistance without and with flow (35 mL/s) were 1.50 ± 0.14 MΩ (n = 3, ± SD) and 1.41 ± 0.10 MΩ (n = 3, ± SD). No significant difference was observed. Moreover, compared to the resistance of external circuit calculated from measured voltage and current in Fig. 4e (68 kΩ), the resistance of the filter was large enough to prevent the leak current. From these results, we concluded that the flow does not influence the electrical resistance.

## Discussion

The maximum performance of the pressure driven electric power generator in this experiment using the foot press unit was 4.8 mW (18 V, 0.26 mA, 0.02% energy efficiency), with a 1.7 s duration when using the filter with 8 μm average pore size that had been sintered at 700 °C. We compared the performance with that of previously reported mechanical energy harvesting devices. Table 1 summarizes them in a comparison of principle, material and performance with a special emphasis on the comparison among devices with the same principle of being water pressure driven.

**Table 1:** Performance comparison of the power generation device with other mechanical energy harvesting devices regarding principle, material (only for the “water pressure driven” generation principle), structure, power, voltage, current, energy efficiency and duration (“n/r” denotes “not reported”).

Principle	Reference	Material	Power	Voltage	Current	Energy efficiency	Duration
Electro-magnetic	^6^	**–**	5.0 W	n/r	n/r	8%	< 1 s
Piezo-electric	^8^	**–**	8.3 mW	4.5 V	1.8 mA	0.03%	< 0.1 s
Electro-static	^14^	**–**	5.0 μW	2.0 V	2.5 μA	70%	< 0.01 s
Water pressure driven	^15^	Silicon (glass coated)	0.55 μW	0.70 V	0.75 μA	2%	n/r
	^16^	Metal–carbon composit	5.6 pW	0.70 mV	80 nA	n/r	> 1 min
	^17^	Wood	3.0 μW	0.30 V	10 μA	n/r	> 1 day
	^18^	Glass (shallow channel)	0.24 nW	1.8 V	0.14 nA	1%	n/r
	^19^		0.20 pW	17 mV	12 pA	5%	n/r
	^20^	Glass (parallel channels)	0.20 μW	0.10 V	2.0 μA	0.04%	n/r
	^21^		36 pW	1.8 V	20 pA	n/r	> 10 min
	^22^	Glass (porous)	20 nW	22 mV	0.92 μW	0.3%	n/r
	This study		4.8 mW	18 V	0.26 mA	0.02%	1.7 s

The electromagnetic device^6^ generates a large power, but it has a large size. Piezoelectric^8^ and electrostatic^14^ devices are rather small, but the duration of energy generation is short. Therefore, it is difficult to use them for motions with a long period (i.e., longer than 1 s). On the other hand, the duration of energy generation of our device is long because generation continues as long as water remains in the device. Among the water pressure based generation approaches, materials other than glass including glass coated silicon^15^, metal–carbon composite^16^, or cellulose^17^ can only provide a small power (less than a few microwatts) because a high pressure cannot be applied due to the fragility of the materials, and therefore the generated voltages are not so high (less than 1 V).

Most of the conventional power generation with the same principle have used the micro or nanochannels fabricated by photolithography^18–21^. The power generation of devices using glass channels made by photolithography is far smaller (less than 1 μW). Even though glass can endure high pressures and therefore higher voltages can be generated than by other materials, the currents are very low (a few microamperes or less). The main objective of these studies is to investigate surface and fluid properties. For this purpose, it is important to define the channel geometry by photolithography, but the current is small due to the difficulty of 3D integration of channels. There is also a report using a sintering glass filter^22^. However, this study used a commercially available filter. This is not optimized for power generation. Therefore, the voltage was low (22 mV), and accordingly the power was still low (20 nW) partly because of the fragility of the porous glass. On the other hand, we have fabricated glass filters from scratch. We have applied the technique for glass-glass bonding at low temperature by applying pressure during sintering process^35,36^, and succeeded in fabricating the robust glass filters usable even at 830 kPa fluid pressure. Therefore, generated power improved over 4 orders of magnitude.

Overall, we fabricated an electric energy generator using a porous glass filter for which we optimized the fabrication process, and we demonstrated power generation for longer than 1 s. This is enough for normal circuit uses such as condensing power by capacitors or boosting voltage, and hence, we demonstrated the usefulness as an energy harvesting generator. This system can be a prototype model for an electric energy generator as an energy source for various human interface devices based on a human mechanical behavior conversion system. This system uses low frequency actuation as the energy source, therefore it is a clean and safe energy supply with many possible future applications.

Additionally, this ion-based generation is basically similar to our previously demonstrated electric ray generator^40^. That generator used electric organs of the ray integrating a number of ion pumps (membrane proteins) passing specific ions using adenosine triphosphate (ATP) as an energy source to transport ions. Our pressure driven electric power generator can be regarded as one of the products inspired from such machine-life fusion devices^41–43^.

## Methods

### Fabrication of the glass filters

Glass filters were fabricated in two ways. In the first, a borosilicate glass frit (Furuuchi Chemical Corporation, Tokyo, Japan) was put in the hole of a carbon mold (Beijing Jinglong Special Carbon Co., Ltd, Beijing, China) (hole diameter of 2 cm and depth of 3 mm) and the frit was sintering. In the second way, a powder of milled glass particles was used. 3 g of coarse particles were milled manually in a mortar (As One, Osaka, Japan), and the rotation speed was constant at 120 rpm. The milling time was monitored by a stop watch. The powder was put into the whole of the carbon mold and then alumina weights (60 g/plate, Yunyi Electronic Co., Ltd, Guangzhou, China) were placed on each particle-filled hole (3 plates/jig) to apply pressure. The glass powder was sintered in a vacuum furnace (KDF-900GL, Denken, Kyoto, Japan) to make the filter. The temperature was raised to the desired temperature (680–720 °C) during 2 h and then maintained for 5 h at it. Then, the mold containing the sinter filter was cooled to room temperature during 8 h. All sintering procedures were carried out in a vacuum condition.

Fused silica filters were fabricated with a similar procedure using SiO_2_ powder (Furuuchi Chemical Corporation). Since the softening temperature of fused silica is quite high, we used the sintering temperature of 1100 °C for 10 h in a vacuum condition for provisional solidification followed by 1150 °C for 5 h for hardening using a non-vacuum furnace (KDF-S80, Denken, Kyoto, Japan) without a carbon mold. Note that the provisional solidification took place in the mold, then the mold was removed and just the filter was hardened using a non-vacuum furnace.

### Mercury porosimetry measurement

The procedure for this has been described in elsewhere^44^. Briefly, the sintered glass filters were broken into small pieces (smaller than 1 cm diameter). Then, they were set in an automatic mercury porosimeter (Pascal 140 for low pressure or Pascal 240 for high pressure; MicrotracBEL, Osaka, Japan). The surface tension and contact angle of mercury used in this measurement were 0.48 N/m and 141.3°, respectively.

### Fabrication of the pressure driven electric power generator

A filter holder (Swinnex Filter Holder Φ25mm, Merck, MA, USA), a luer fitting (VPRM406, ISIS, Osaka, Japan), a copper mesh sheet (#100, Eggs Store, Tokyo, Japan) and silicone tubes were prepared. The outlet of the filter holder was shaved using a hobby router (HRT-86, Takagi, Niigata, Japan) to expand the opening. The luer fitting was attached to the top of the filter holder and connected to the silicone tube (4 mm diameter). The bottom of the holder was attached to another silicone tube (10 mm diameter). Two circles (diameter of 20 mm) were cut from the copper mesh, and one circle was put on top and of the other underneath the filter for the electrodes. Finally, conducting wires were attached to the electrodes by solder through holes pierced in the silicone tubes.

### Setup of the water delivery system

Water was introduced from a 50 mL syringe (SS-50ESZ, Terumo, Tokyo, Japan) into the power generator at a constant speed by a linear actuator. The speed of the linear actuator (LEY32C-150, SMC, Tokyo, Japan) was controlled by an ACT controller (version 1.2.0.0, SMC). Water was circulated in one direction by pushing and pulling motions of the syringe piston by the linear actuator via a check valve (AS-1022, ASOH, Osaka, Japan).

### Measurement of voltage, current and force

Signals of the voltage, current and force were sent to a PC through an I/O board (MF644, Humusoft, Prague, Czech Republic). with MATLAB R2020a (version 9.9.01467702, MathWorks, MA, USA) and Simulink (version 10.2, MathWorks) were installed on the PC. A strain gauge sensor (LSM-50K-B, MinebeaMitsumi, Nagano, Japan) was set on top of the syringe piston. The force signal was also sent to the I/O board via a strain amplifier (DPM-951A, Kyowa Electronic Instruments, Tokyo, Japan). A voltage divider using 1 MΩ with 2, 5 and 10 MΩ serially connected resistances which were selected according to the measured voltage range was employed. The current was calculated by the slope of the voltage stored in a capacitor (200 μF) during the experimenter’s foot pressing. For the for press unit, a data logger (NR-600, Keyence, Osaka, Japan) with a high-speed analog measurement system (NR-HA08, Keyence) and software (WAVE LOGGER PRO (version R4.02.00), Keyence) were used for recording. The data were recorded at 1 ms time intervals.

### Fabrication of the foot press unit.

We designed and fabricated a large foot pressing power actuated unit (Fig. 4b) for demonstration of the power generation. The unit had a syringe holder (59 mm in diameter) and a syringe (31 mm in diameter). It also had a square cover (100 mm × 100 mm) with an inner molded cylinder shape (85 mm in diameter into which the syringe holder fit) to enlarge the force application area and limit the working directions, and an inner cylindrical stand (34 mm in diameter) to keep the whole unit stable. The components of the unit was fabricated by using a 3D fused deposition modeling (FDM) printer (Black knight, Magic maker, Chongqing, China). The material used for this unit was polylactic acid (PLA). The design was drawn using design software (Fusion 360, Autodesk, CA, USA).

### Preparation of electronic devices for application tests

A LED light (3 mm, 3.3–3.6 V, 18 mA) was used for the illumination test and no resistance was used. The stored energy in a capacitor was measured with a multimeter (MS8233D, Crenova, China). For the mini fan rotation, a mini motor (DC 1.5–3 V/40 mA, 11 × 4 mm, Uxcell Micro Vibrating Motor (OEM), Hong Kong, China) and other 3D printed parts were used. For the wireless communication tool test, a signal generator (STM320, EnOcean, Oberhaching, Germany) was used with a signal receiver (USB 400 J, EnOcean) and a wireless kit (ESK300U, EnOcean).

### Image capture and analysis

The SEM images of glass particles were acquired by a scanning electron microscope (VE-8800, Keyence) and analyzed using ImageJ (version 1.8.0_172)^45^ open-source software. The average Feret diameters were automatically measured by the software after the image data were binarized as white–black. The acquired videos and images for application experiments were edited by commercial software (PowerDirector 16 (version 16.0), CyberLink, New Taipei City, Taiwan).

### Measurement and simulation of electrical resistance in a glass filter

The static resistance of the filter was measured by using a high accuracy multimeter (Fluke 83 multimeter, Washington, USA). In flow condition, a simple circuit was built to stabilize the current and separate the total generated voltage to a reference resistance (2.05 MΩ). This is because it is difficult to measure the resistance by using a multimeter directly. According to the simple Ohm's law, the resistance at flow condition was estimated. Numerical simulation was carried using AC and CFD modules of commercial software (COMSOL Multiphysics (version 6.0), COMSOL Inc., Burlington, MA, USA).

## Supplementary Information

Supplementary Figures.

Supplementary Movie 1.

Supplementary Movie 2.

Supplementary Movie 3.

## Data Availability

The authors declare that all data supporting the findings of this study are available within the paper and the Supplementary Information.
