# Supplementary material for: A pressure driven electric energy generator exploiting a micro- to nano-scale glass porous filter with ion flow originating from water
Source: Sci Rep. 2022 Oct 20;12:16827. doi: 10.1038/s41598-022-21069-8 (PMC9585039; doi:10.1038/s41598-022-21069-8)
Supplement: Supplementary file 1 — Supplementary Figures. [file 41598_2022_21069_MOESM1_ESM.pdf]

Supporting Information

A pressure driven electric energy generator exploiting a micro- to nano-scale glass porous filter with ion flow originating from water

Yo Tanaka*[1], Satoshi Amaya[1], Shun-ichi Funano[1], Hisashi Sugawa[2], Wataru Nagafuchi[2], Yuri Ito[1], Yusufu Aishan[1], Xun Liu[3], Norihiro Kamamichi[2] & Yaxiaer Yalikun[1,3]

[1] Center for Biosystems Dynamics Research (BDR), RIKEN, 1-3 Yamadaoka, Suita, Osaka 565-0871, Japan

[2] Department of Robotics and Mechatronics, Tokyo Denki University, 5 Senju-asahi-cho, Adachi-ku, Tokyo 120-8551, Japan

[3] Graduate School of Nara Institute of Science and Technology, 8916-5 Takayamacho, Ikoma, Nara 630-0192, Japan

*To whom correspondence should be addressed: E-mail: yotanaka1980@gmail.com

TEL: +81-6-6105-5132, FAX: +81-6-6105-5132

Table of Contents:

- **Supplementary Figures (Figs. S1-S8)**
- **Supplementary Movie Captions (Movies 1-3)**

Supplementary Figures and Legends

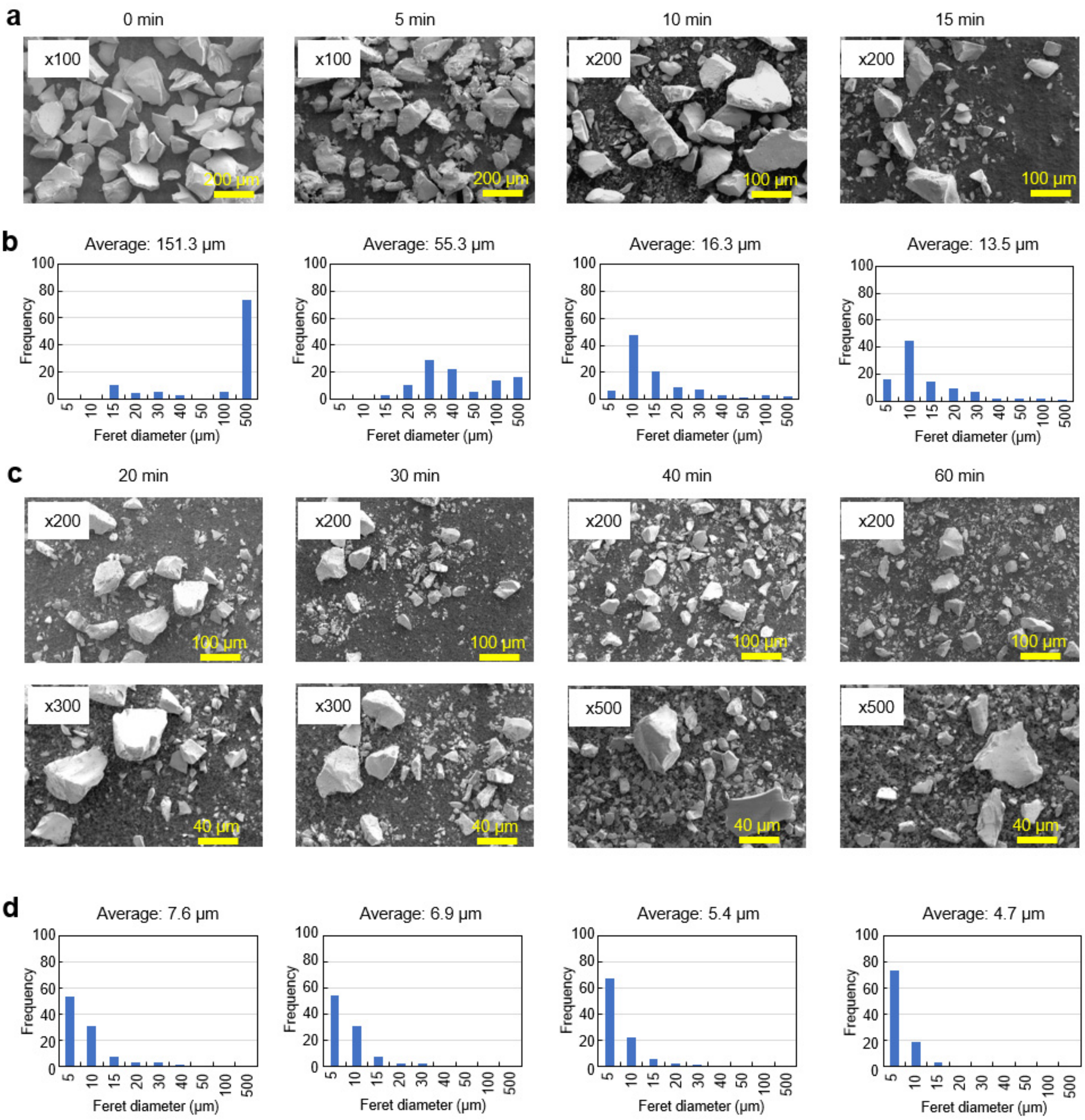

Figure S1 Image analysis of ground glass powders. (**a**) SEM images of the milled glass powder with the milling time indicated at the top of each image. (**b**) Histogram of particle Feret diameter by analyzing 100 particles; the average

Feret diameter is indicated at the top of each graph. (**c**) Higher magnification SEM images of powder with longer milling time than in (**a**). Scale bars are shown in yellow. (**d**) Histogram of particle Feret diameter corresponding to images of (**c**). The histogram was obtained by analyzing 100 particles.

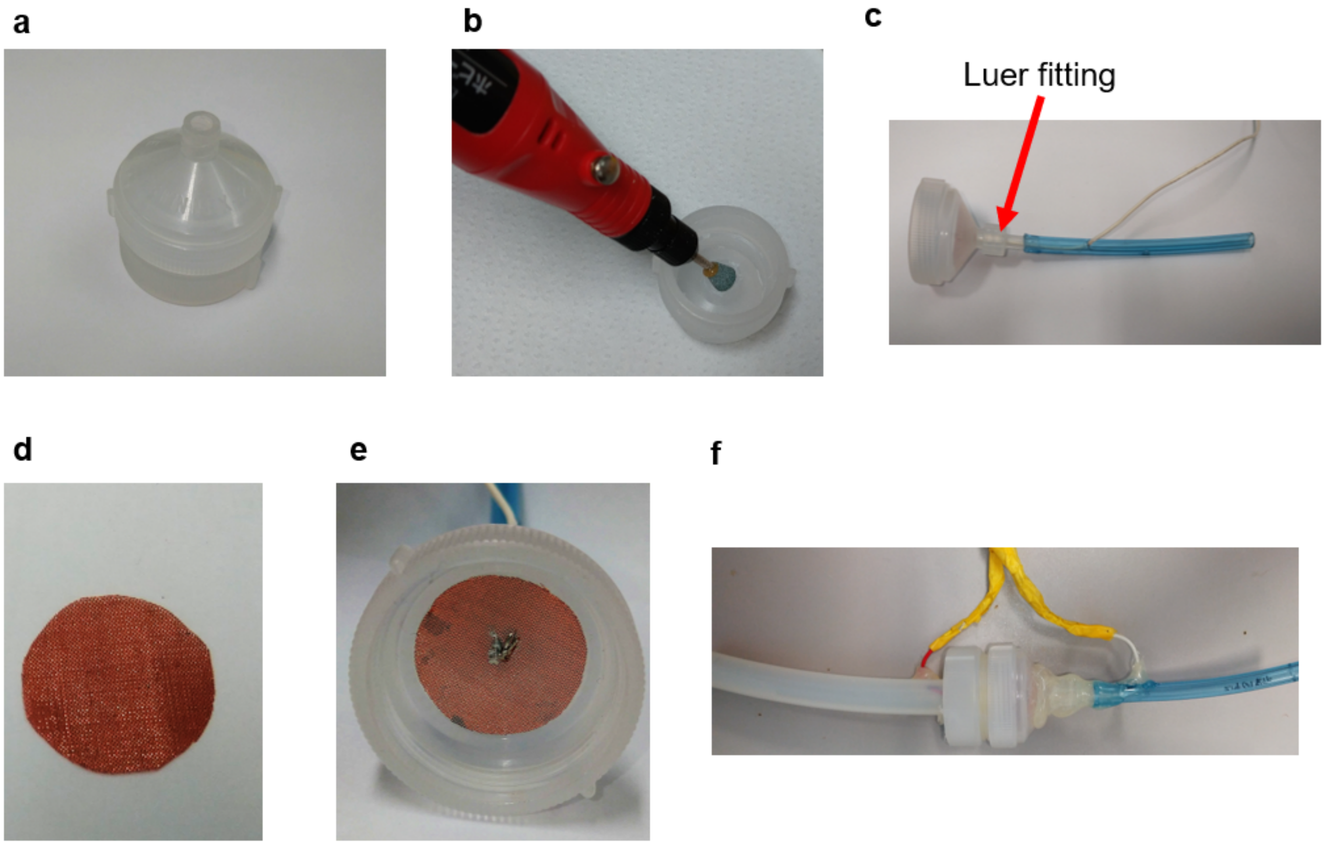

Figure S2 Photos of materials and the fabrication process of a generator without the glass filter. (**a**) Holder of the generator. (**b**) Expanding a hole at the bottom of the holder using a hand router. (**c**) Holder connected with a tube at the inlet port by a luer fitting. (**d**) Copper mesh electrode (#100) cut to fit the holder. (**e**) Inside of the holder equipped with the mesh electrode and solder connections. (**f**) A completed generator.

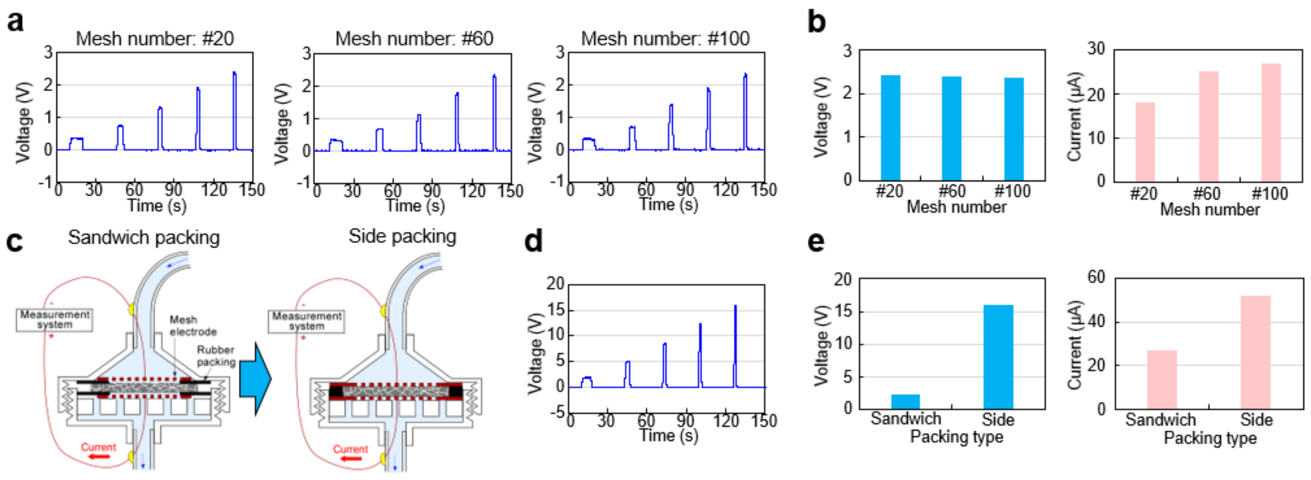

Figure S3 **Experimental results for optimization of the generator.** (**a**) Voltage time-courses using the mesh number indicated at the top of each graph. The glass filter sintered at 700ºC without milling was used. Each graph shows voltage during 1 pressing cycle at the water delivery system speed of 2, 4, 6, 8, and 10 mm/s. (**b**) Voltage and current at 10 mm/s speed using different mesh numbers. (**c**) Comparison of generators installed with 2 types of packings (sandwich and side). Sandwich type packing was used in the measurement for (**a**) and (**b**). (**d**) Voltage time-course using the side packing. The glass filter sintered at 700ºC without milling and #100 mesh was used. The graph shows voltage during 1 pressing cycle at the water delivery system speed of 2, 4, 6, 8, and 10 mm/s. (**e**) Voltage and current at 10 mm/s speed using 2 types of packings.

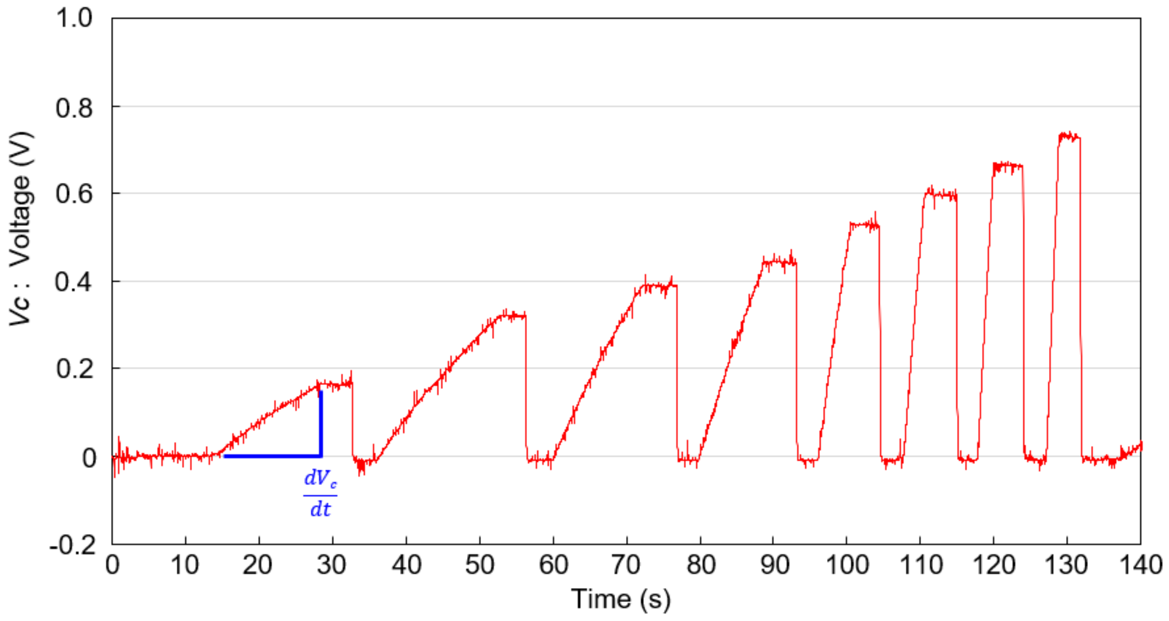

Figure S4 Raw data of current measurement using a capacitor and an explanation of the estimation. The graph shows a time-course of stored voltage in a capacitor using the glass filter sintered at 700ºC without milling. It shows voltage during 1 pressing cycle at water delivery system speed of 4, 6, 8, 10, 20, 30, 40 and 50 mm/s(corresponding to the graph of 1 cycle of Fig. 3e, 700ºC). After each pressing cycle, the voltage in the capacitor was released. From the slope during the pressing (*dVc/dt*), the current can be calculated by multiplying by *C* (capacity of the capacitor).

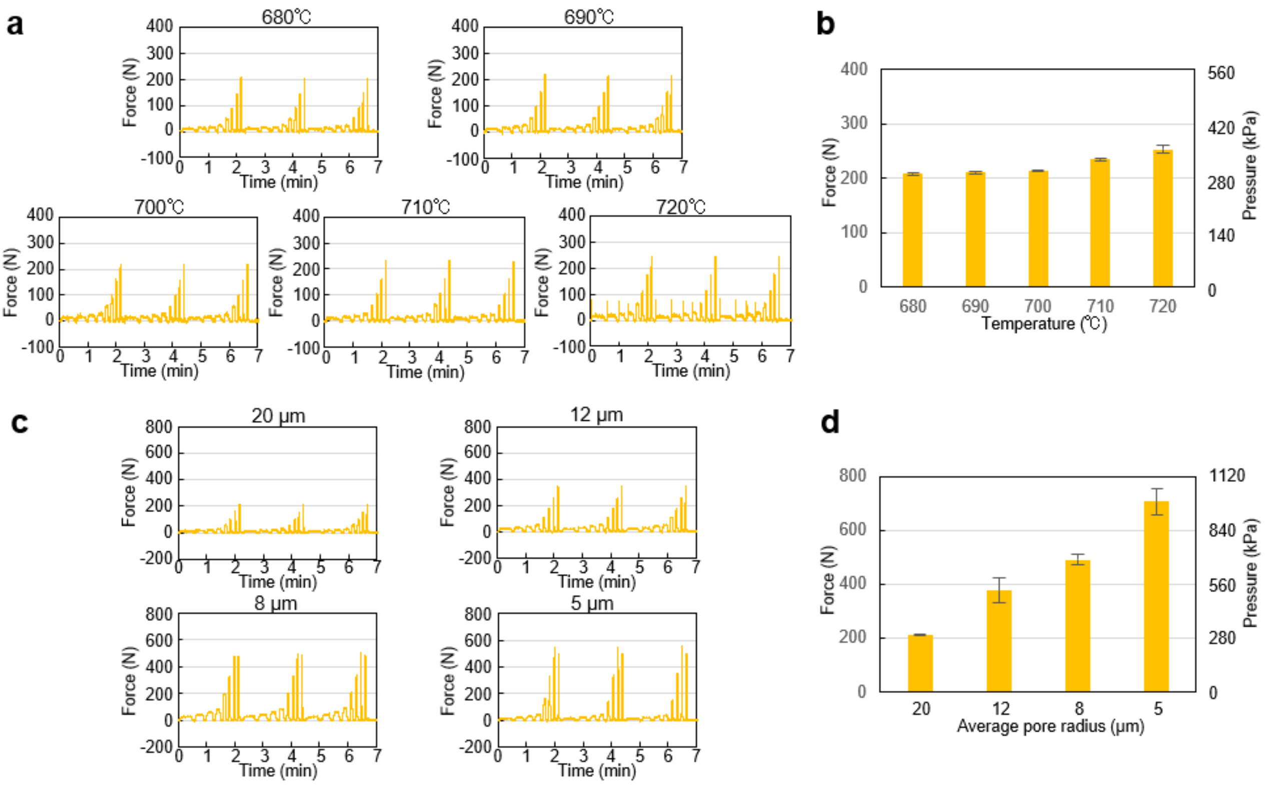

Figure S5 **Measured force using the porous glass filters.** (**a**) Force time-courses during the voltage measurement using filters sintered at various temperatures indicated at the top of each graph. (**b**) Force and corresponding pressure applied to the generator vs. sintering temperature of the glass filters at the water delivery system speed of 50 mm/s. Plots represent average ± S.D. (n=3). (**c**) Force time-courses during the voltage measurement using filters sintered at 700ºC at various average pore radii indicated at the top of each graph. (**d**) Force and corresponding pressure applied to the generators versus average pore radius of the glass filters at the water delivery system speed of 50 mm/s. Pressure was calculated by dividing the force by the area of syringe cross-section (31 mm diameter). Plots represent average ± S.D. (n=3). (**a**) and (**c**) correspond to Figs. 3 (**e**)

and (**g**), respectively. Each graph shows force during 3 pressing cycles at the water delivery system speed of 4, 6, 8, 10, 20, 30, 40 and 50 mm/s. Voltage and force were measured simultaneously.

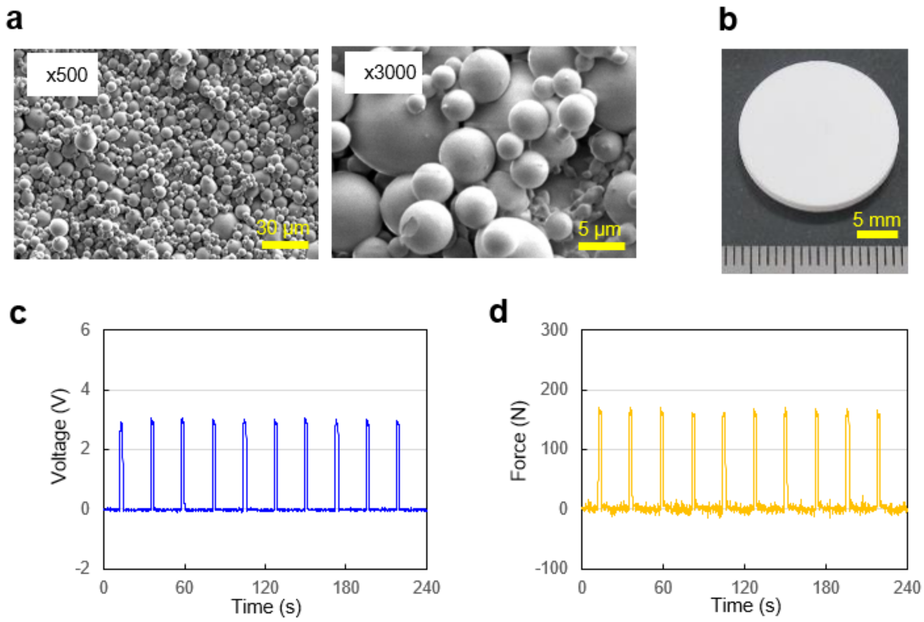

Figure S6 Verification experiment of the generator using a filter made with fused silica powder. (**a**) SEM images of the porous fused silica filter surface sintered at 1100ºC. (**b**) A sintered porous fused silica filter. (**c**) Voltage and (**d**) force time-courses of the repetitive power generation using the fused silica filter at the water delivery system speed of 40 mm/s. Scale bars are shown in yellow in (**a**) and (**b**).

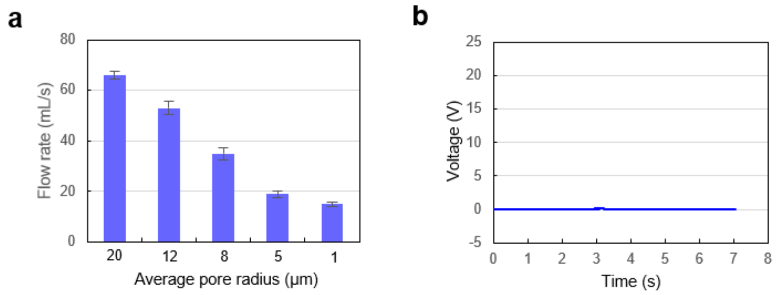

Figure S7 **Additional validation data.** (**a**) Flow rate versus average pore radius in foot-press experiment. Plots represent average ± S.D. (n=3). (**b**) Voltage time-course by foot-press without a glass filter. Press started between 2 and 3 s.

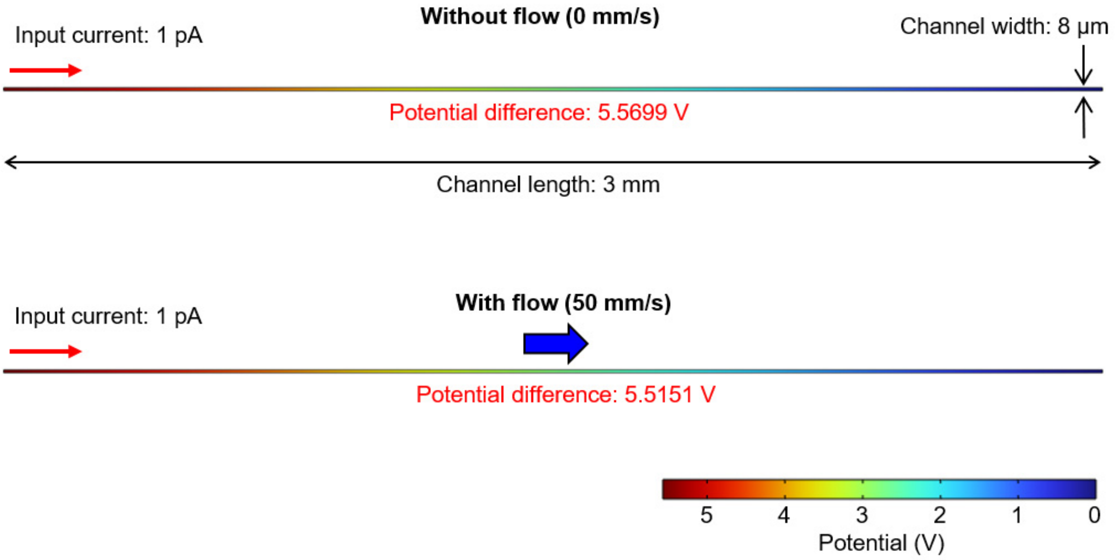

Figure S8 Simulation result showing distribution of potential to estimate the resistance change with or without flow in a microchannel. The channel in simulation is 3 mm long 8 μm width and depth resembling the actual filter channel. The input of current was set as 1 pA and output potential was set as ground (0 V). Flow velocity is set as 50 mm/s. Water relative permittivity and electrical resistivity was set as 80 and 18.2 MΩ cm, respectively. The potential differences between the channel inlet and outlet without and with flow were 5.5699 and 5.5151 V, respectively. The resistances without and with flow were 5.5699 and 5.5151 TΩ, respectively. Note that the simulated resistance was much higher than the actually measured value (about 1.50 MΩ), because this simulation dealt with just single channel differently from the pore glass filter.

Supplementary Movie Legends

Movie 1 | This movie shows real-time direct LED lighting by the current produced from the pure water flow through the generator equipped with a foot press unit. The LED was bright during the foot pressing. The experiment corresponds to Fig. 4f. Inset: Circuit status during the experiment.

Movie 2 | This movie shows real-time driving of a rotator by stored energy in a capacitor. The energy was accumulated in the capacitor for 50 cycles of foot pressing and after that the multimeter display screen showed 5 V accumulation in the capacitor. By turning the switch on to release the energy in the capacitor, the rotator was driven for a few tenths of a second. A slow motion version of the rotation (1/10 speed) is added to clearly show the rotation. The experiment corresponds to Fig. 4g. Inset: Circuit status during the experiment

Movie 3 | This movie shows real-time wireless communication by stored energy in a capacitor. The energy was accumulated in the capacitor by foot pressing. Immediately after the energy accumulation, the stored energy was automatically released to drive the signal sender, and the screen shows the recipient of 3 signals. The experiment corresponds to Fig. 4h. Inset: Circuit status during the experiment.
